# Supplementary material for: Deletion of wfs1 Impairs Oligodendrocyte Precursor Cells Dorsal Distribution and Myelination Through the wfs1-hmgcs1 Axis in Zebrafish
Source: Biology (Basel). 2026 Aug 16;15(16):1402. doi: 10.3390/biology15161402 (PMC13510151; doi:10.3390/biology15161402)
Supplement: Supplementary file 1 [file biology-15-01402-s001.zip › File S1-Transcriptome sequencing data and differentially expressed genes (DEGs) in wfs1-- zebrafish at 5 dpf/2.KEGG/wfs1KOvsWT/DOWN/src/dre04060.html]

dre04060


- dre:562247

- Down regulated genes

562247\_1(-1.84336770590498)

- dre:799354

- Down regulated genes

799354\_1(-1.21394100025012)

- dre:100002946

- Down regulated genes

100002946(-1.40391573648291)

- dre:100037357

- Down regulated genes

100037357(-1.39102944946719)

- dre:100006078

- Down regulated genes

100006078\_1(-5.72416507735977)

- dre:368326

- Down regulated genes

368326(-1.63536295102477)

- dre:368326

- Down regulated genes

368326(-1.63536295102477)

Close
